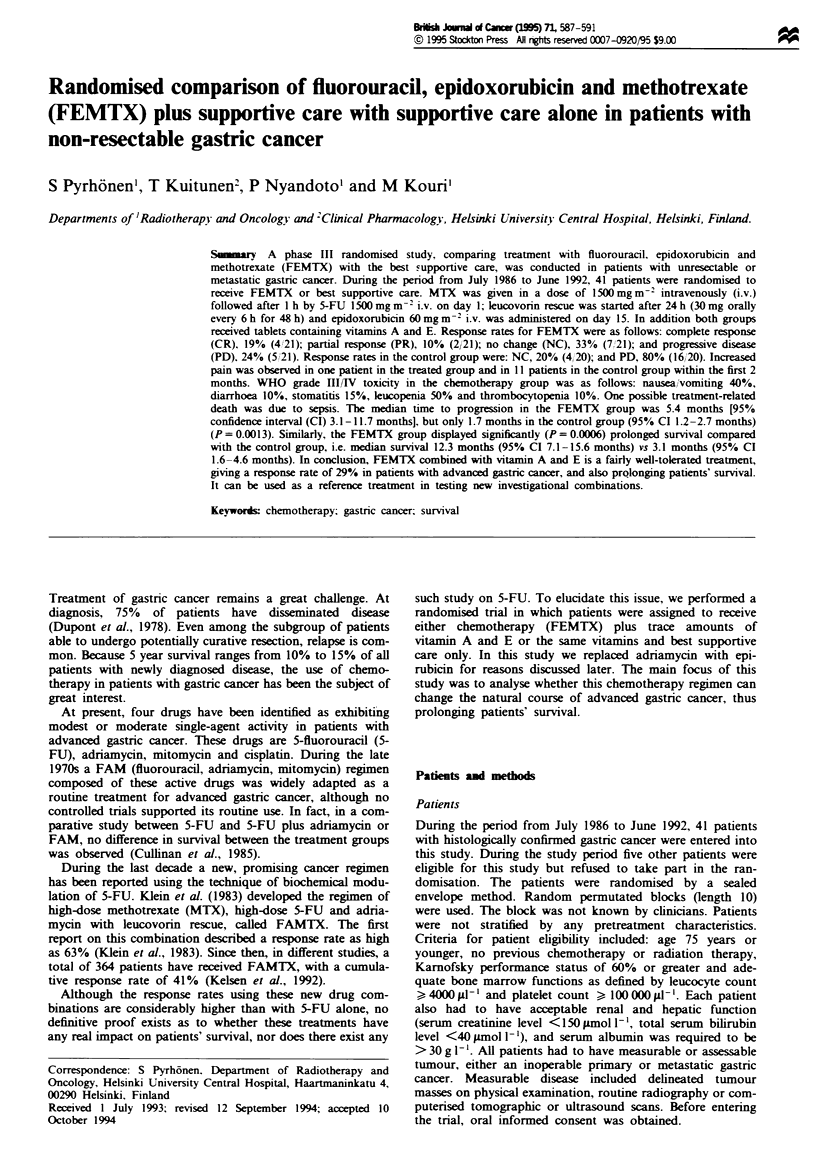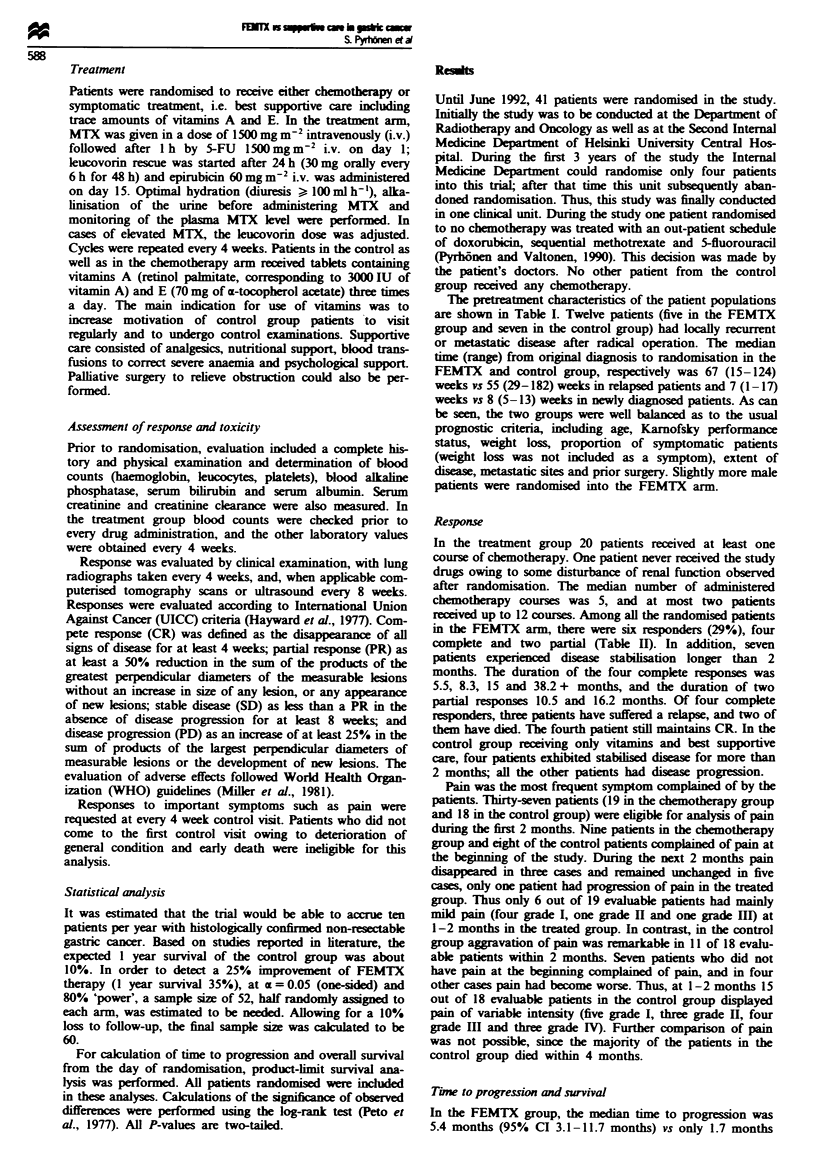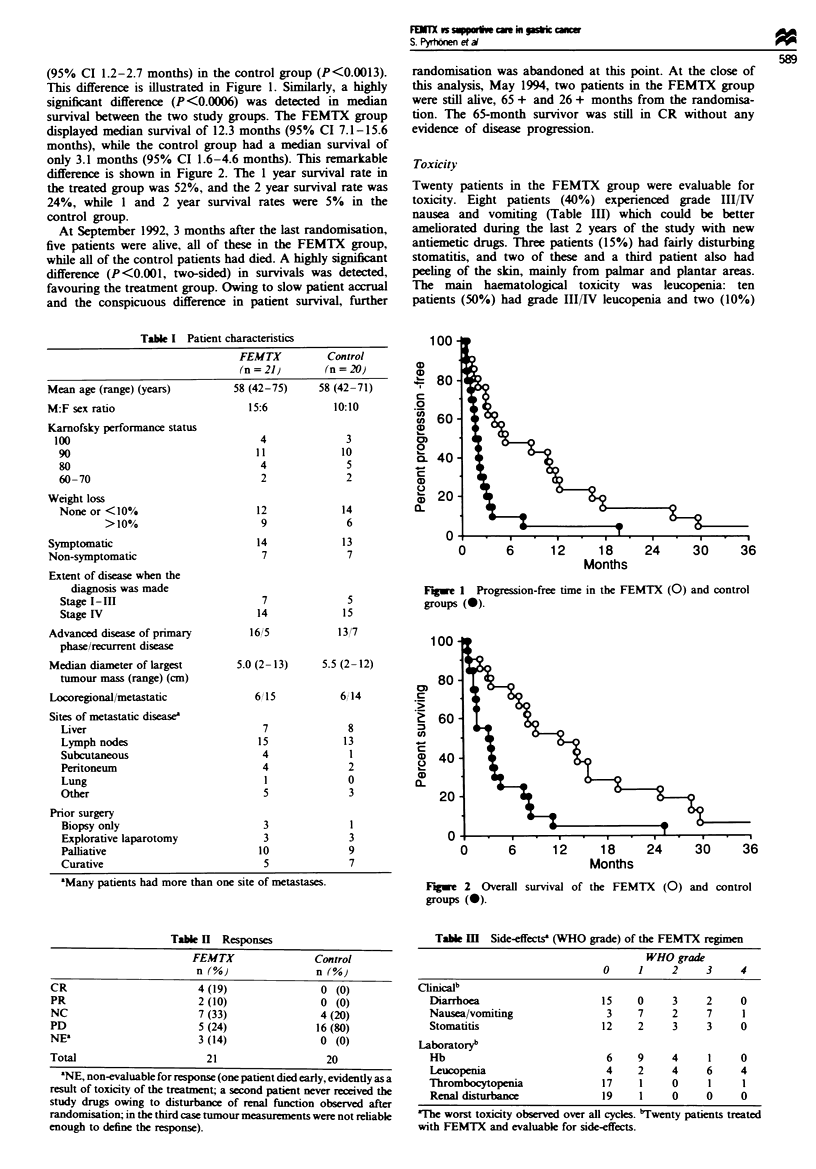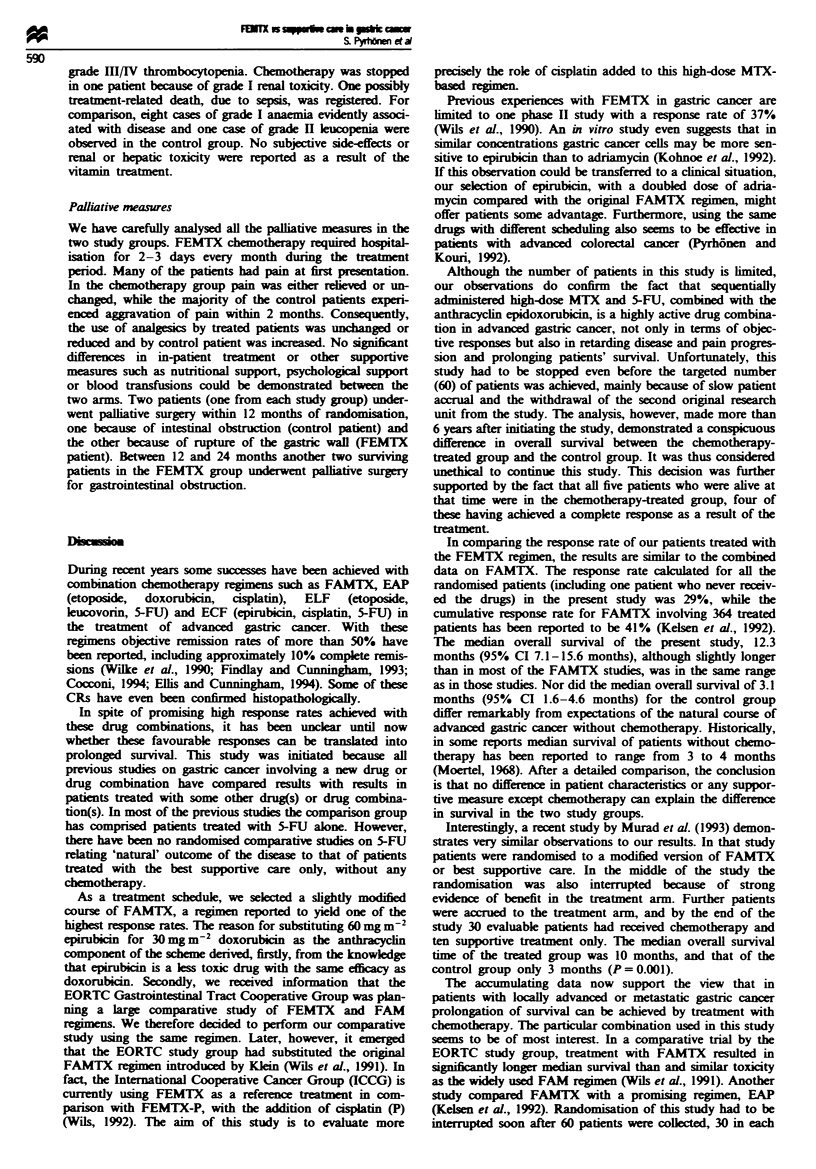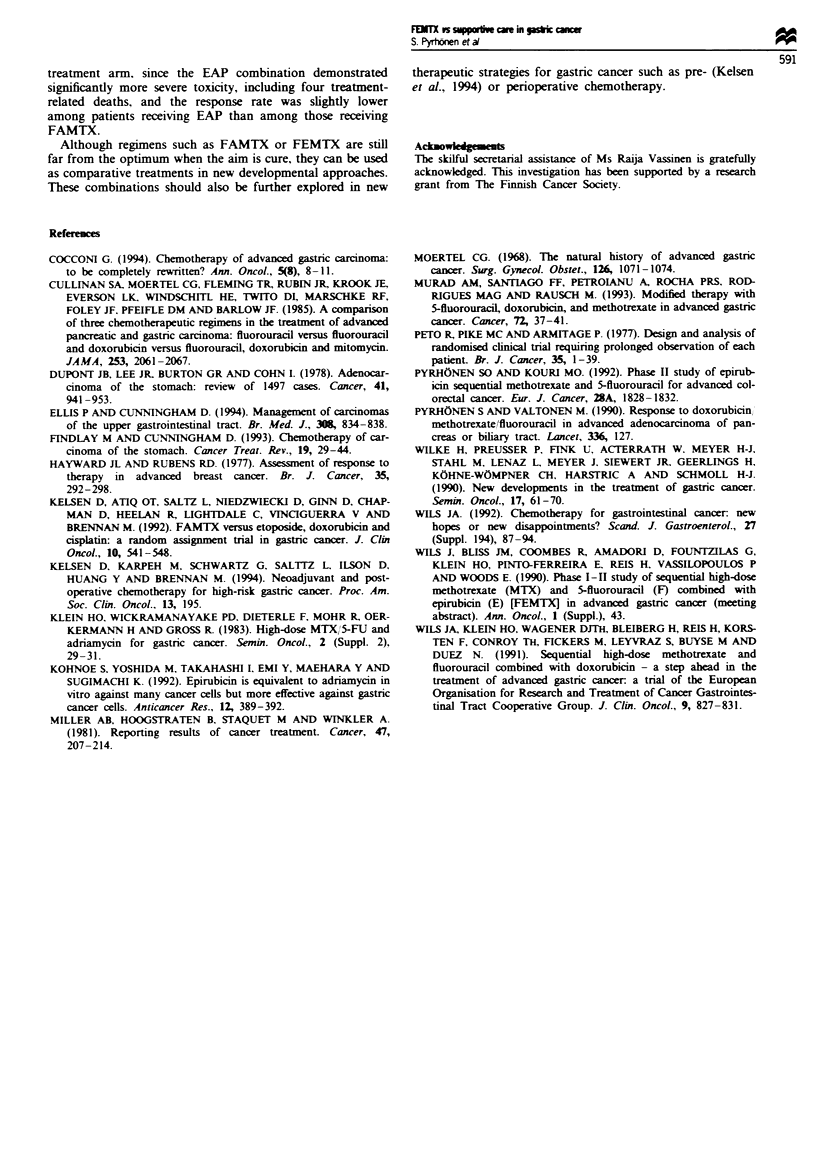# Randomised comparison of fluorouracil, epidoxorubicin and methotrexate (FEMTX) plus supportive care with supportive care alone in patients with non-resectable gastric cancer.

**DOI:** 10.1038/bjc.1995.114

**Published:** 1995-03

**Authors:** S. Pyrhönen, T. Kuitunen, P. Nyandoto, M. Kouri

**Affiliations:** Department of Radiotherapy and Oncology, Helsinki University Central Hospital, Finland.

## Abstract

A phase III randomised study, comparing treatment with fluorouracil, epidoxorubicin and methotrexate (FEMTX) with the best supportive care, was conducted in patients with unresectable or metastatic gastric cancer. During the period from July 1986 to June 1992, 41 patients were randomised to receive FEMTX or best supportive care. MTX was given in a dose of 1500 mg m-2 intravenously (i.v.) followed after 1 h by 5-FU 1500 mg m-2 i.v. on day 1; leucovorin rescue was started after 24 h (30 mg orally every 6 h for 48 h) and epidoxorubicin 60 mg m-2 i.v. was administered on day 15. In addition both groups received tablets containing vitamins A and E. Response rates for FEMTX were as follows: complete response (CR), 19% (4/21); partial response (PR), 10% (2/21); no change (NC), 33% (7/21); and progressive disease (PD), 24% (5/21). Response rates in the control group were: NC, 20% (4/20); and PD, 80% (16/20). Increased pain was observed in one patient in the treated group and in 11 patients in the control group within the first 2 months. WHO grade III/IV toxicity in the chemotherapy group was as follows: nausea/vomiting 40%, diarrhoea 10%, stomatitis 15%, leucopenia 50% and thrombocytopenia 10%. One possible treatment-related death was due to sepsis. The median time to progression in the FEMTX group was 5.4 months [95% confidence interval (CI) 3.1-11.7 months], but only 1.7 months in the control group (95% CI 1.2-2.7 months) (P = 0.0013). Similarly, the FEMTX group displayed significantly (P = 0.0006) prolonged survival compared with the control group, i.e. median survival 12.3 months (95% CI 7.1-15.6 months) vs 3.1 months (95% CI 1.6-4.6 months). In conclusion, FEMTX combined with vitamin A and E is a fairly well-tolerated treatment, giving a response rate of 29% in patients with advanced gastric cancer, and also prolonging patients' survival. It can be used as a reference treatment in testing new investigational combinations.


© 1995 Stockton Press All rights reserved 0007-0920/95 $9.00

### Randomised comparison of fluorouracil, epidoxorubicin and methotrexate (FEMTX) plus supportive care with supportive care alone in patients with non-resectable gastric cancer

S Pyrhönen[1], T Kuitunen[2], P Nyandoto[1] and M Kouri[1]

*Departments of [1]Radiotherapy and Oncology and [2]Clinical Pharmacology, Helsinki University Central Hospital, Helsinki, Finland.*

**Summary** A phase III randomised study, comparing treatment with fluorouracil, epidoxorubicin and methotrexate (FEMTX) with the best supportive care, was conducted in patients with unresectable or metastatic gastric cancer. During the period from July 1986 to June 1992, 41 patients were randomised to receive FEMTX or best supportive care. MTX was given in a dose of 1500 mg $m^{-2}$ intravenously (i.v.) followed after 1 h by 5-FU 1500 mg $m^{-2}$ i.v. on day 1; leucovorin rescue was started after 24 h (30 mg orally every 6 h for 48 h) and epidoxorubicin 60 mg $m^{-2}$ i.v. was administered on day 15. In addition both groups received tablets containing vitamins A and E. Response rates for FEMTX were as follows: complete response (CR), 19% (4/21); partial response (PR), 10% (2/21); no change (NC), 33% (7/21); and progressive disease (PD), 24% (5/21). Response rates in the control group were: NC, 20% (4/20); and PD, 80% (16/20). Increased pain was observed in one patient in the treated group and in 11 patients in the control group within the first 2 months. WHO grade III/IV toxicity in the chemotherapy group was as follows: nausea/vomiting 40%, diarrhoea 10%, stomatitis 15%, leucopenia 50% and thrombocytopenia 10%. One possible treatment-related death was due to sepsis. The median time to progression in the FEMTX group was 5.4 months [95% confidence interval (CI) 3.1-11.7 months], but only 1.7 months in the control group (95% CI 1.2-2.7 months) ($P = 0.0013$). Similarly, the FEMTX group displayed significantly ($P = 0.0006$) prolonged survival compared with the control group, i.e. median survival 12.3 months (95% CI 7.1-15.6 months) *vs* 3.1 months (95% CI 1.6-4.6 months). In conclusion, FEMTX combined with vitamin A and E is a fairly well-tolerated treatment, giving a response rate of 29% in patients with advanced gastric cancer, and also prolonging patients' survival. It can be used as a reference treatment in testing new investigational combinations.

**Keywords:** chemotherapy; gastric cancer; survival

Treatment of gastric cancer remains a great challenge. At diagnosis, 75% of patients have disseminated disease (Dupont *et al.*, 1978). Even among the subgroup of patients able to undergo potentially curative resection, relapse is common. Because 5 year survival ranges from 10% to 15% of all patients with newly diagnosed disease, the use of chemotherapy in patients with gastric cancer has been the subject of great interest.

At present, four drugs have been identified as exhibiting modest or moderate single-agent activity in patients with advanced gastric cancer. These drugs are 5-fluorouracil (5-FU), adriamycin, mitomycin and cisplatin. During the late 1970s a FAM (fluorouracil, adriamycin, mitomycin) regimen composed of these active drugs was widely adapted as a routine treatment for advanced gastric cancer, although no controlled trials supported its routine use. In fact, in a comparative study between 5-FU and 5-FU plus adriamycin or FAM, no difference in survival between the treatment groups was observed (Cullinan *et al.*, 1985).

During the last decade a new, promising cancer regimen has been reported using the technique of biochemical modulation of 5-FU. Klein *et al.* (1983) developed the regimen of high-dose methotrexate (MTX), high-dose 5-FU and adriamycin with leucovorin rescue, called FAMTX. The first report on this combination described a response rate as high as 63% (Klein *et al.*, 1983). Since then, in different studies, a total of 364 patients have received FAMTX, with a cumulative response rate of 41% (Kelsen *et al.*, 1992).

Although the response rates using these new drug combinations are considerably higher than with 5-FU alone, no definitive proof exists as to whether these treatments have any real impact on patients' survival, nor does there exist any such study on 5-FU. To elucidate this issue, we performed a randomised trial in which patients were assigned to receive either chemotherapy (FEMTX) plus trace amounts of vitamin A and E or the same vitamins and best supportive care only. In this study we replaced adriamycin with epirubicin for reasons discussed later. The main focus of this study was to analyse whether this chemotherapy regimen can change the natural course of advanced gastric cancer, thus prolonging patients' survival.

Correspondence: S Pyrhönen, Department of Radiotherapy and Oncology, Helsinki University Central Hospital, Haartmaninkatu 4, 00290 Helsinki, Finland
Received 1 July 1993; revised 12 September 1994; accepted 10 October 1994

### Patients and methods

### *Patients*

During the period from July 1986 to June 1992, 41 patients with histologically confirmed gastric cancer were entered into this study. During the study period five other patients were eligible for this study but refused to take part in the randomisation. The patients were randomised by a sealed envelope method. Random permutated blocks (length 10) were used. The block was not known by clinicians. Patients were not stratified by any pretreatment characteristics. Criteria for patient eligibility included: age 75 years or younger, no previous chemotherapy or radiation therapy, Karnofsky performance status of 60% or greater and adequate bone marrow functions as defined by leucocyte count $\geqslant 4000\ \mu l^{-1}$ and platelet count $\geqslant 100\,000\ \mu l^{-1}$. Each patient also had to have acceptable renal and hepatic function (serum creatinine level $< 150\ \mu mol\ l^{-1}$, total serum bilirubin level $< 40\ \mu mol\ l^{-1}$), and serum albumin was required to be $> 30\ g\ l^{-1}$. All patients had to have measurable or assessable tumour, either an inoperable primary or metastatic gastric cancer. Measurable disease included delineated tumour masses on physical examination, routine radiography or computerised tomographic or ultrasound scans. Before entering the trial, oral informed consent was obtained.

### Treatment

Patients were randomised to receive either chemotherapy or symptomatic treatment, i.e. best supportive care including trace amounts of vitamins A and E. In the treatment arm, MTX was given in a dose of 1500 mg $m^{-2}$ intravenously (i.v.) followed after 1 h by 5-FU 1500 mg $m^{-2}$ i.v. on day 1; leucovorin rescue was started after 24 h (30 mg orally every 6 h for 48 h) and epirubicin 60 mg $m^{-2}$ i.v. was administered on day 15. Optimal hydration (diuresis $\geqslant$ 100 ml $h^{-1}$), alkalinisation of the urine before administering MTX and monitoring of the plasma MTX level were performed. In cases of elevated MTX, the leucovorin dose was adjusted. Cycles were repeated every 4 weeks. Patients in the control as well as in the chemotherapy arm received tablets containing vitamins A (retinol palmitate, corresponding to 3000 IU of vitamin A) and E (70 mg of $\alpha$-tocopherol acetate) three times a day. The main indication for use of vitamins was to increase motivation of control group patients to visit regularly and to undergo control examinations. Supportive care consisted of analgesics, nutritional support, blood transfusions to correct severe anaemia and psychological support. Palliative surgery to relieve obstruction could also be performed.

### Assessment of response and toxicity

Prior to randomisation, evaluation included a complete history and physical examination and determination of blood counts (haemoglobin, leucocytes, platelets), blood alkaline phosphatase, serum bilirubin and serum albumin. Serum creatinine and creatinine clearance were also measured. In the treatment group blood counts were checked prior to every drug administration, and the other laboratory values were obtained every 4 weeks.

Response was evaluated by clinical examination, with lung radiographs taken every 4 weeks, and, when applicable computerised tomography scans or ultrasound every 8 weeks. Responses were evaluated according to International Union Against Cancer (UICC) criteria (Hayward *et al.*, 1977). Compete response (CR) was defined as the disappearance of all signs of disease for at least 4 weeks; partial response (PR) as at least a 50% reduction in the sum of the products of the greatest perpendicular diameters of the measurable lesions without an increase in size of any lesion, or any appearance of new lesions; stable disease (SD) as less than a PR in the absence of disease progression for at least 8 weeks; and disease progression (PD) as an increase of at least 25% in the sum of products of the largest perpendicular diameters of measurable lesions or the development of new lesions. The evaluation of adverse effects followed World Health Organization (WHO) guidelines (Miller *et al.*, 1981).

Responses to important symptoms such as pain were requested at every 4 week control visit. Patients who did not come to the first control visit owing to deterioration of general condition and early death were ineligible for this analysis.

### Statistical analysis

It was estimated that the trial would be able to accrue ten patients per year with histologically confirmed non-resectable gastric cancer. Based on studies reported in literature, the expected 1 year survival of the control group was about 10%. In order to detect a 25% improvement of FEMTX therapy (1 year survival 35%), at $\alpha = 0.05$ (one-sided) and 80% 'power', a sample size of 52, half randomly assigned to each arm, was estimated to be needed. Allowing for a 10% loss to follow-up, the final sample size was calculated to be 60.

For calculation of time to progression and overall survival from the day of randomisation, product-limit survival analysis was performed. All patients randomised were included in these analyses. Calculations of the significance of observed differences were performed using the log-rank test (Peto *et al.*, 1977). All *P*-values are two-tailed.

### Results

Until June 1992, 41 patients were randomised in the study. Initially the study was to be conducted at the Department of Radiotherapy and Oncology as well as at the Second Internal Medicine Department of Helsinki University Central Hospital. During the first 3 years of the study the Internal Medicine Department could randomise only four patients into this trial; after that time this unit subsequently abandoned randomisation. Thus, this study was finally conducted in one clinical unit. During the study one patient randomised to no chemotherapy was treated with an out-patient schedule of doxorubicin, sequential methotrexate and 5-fluorouracil (Pyrhönen and Valtonen, 1990). This decision was made by the patient's doctors. No other patient from the control group received any chemotherapy.

The pretreatment characteristics of the patient populations are shown in Table I. Twelve patients (five in the FEMTX group and seven in the control group) had locally recurrent or metastatic disease after radical operation. The median time (range) from original diagnosis to randomisation in the FEMTX and control group, respectively was 67 (15–124) weeks *vs* 55 (29–182) weeks in relapsed patients and 7 (1–17) weeks *vs* 8 (5–13) weeks in newly diagnosed patients. As can be seen, the two groups were well balanced as to the usual prognostic criteria, including age, Karnofsky performance status, weight loss, proportion of symptomatic patients (weight loss was not included as a symptom), extent of disease, metastatic sites and prior surgery. Slightly more male patients were randomised into the FEMTX arm.

### Response

In the treatment group 20 patients received at least one course of chemotherapy. One patient never received the study drugs owing to some disturbance of renal function observed after randomisation. The median number of administered chemotherapy courses was 5, and at most two patients received up to 12 courses. Among all the randomised patients in the FEMTX arm, there were six responders (29%), four complete and two partial (Table II). In addition, seven patients experienced disease stabilisation longer than 2 months. The duration of the four complete responses was 5.5, 8.3, 15 and 38.2+ months, and the duration of two partial responses 10.5 and 16.2 months. Of four complete responders, three patients have suffered a relapse, and two of them have died. The fourth patient still maintains CR. In the control group receiving only vitamins and best supportive care, four patients exhibited stabilised disease for more than 2 months; all the other patients had disease progression.

Pain was the most frequent symptom complained of by the patients. Thirty-seven patients (19 in the chemotherapy group and 18 in the control group) were eligible for analysis of pain during the first 2 months. Nine patients in the chemotherapy group and eight of the control patients complained of pain at the beginning of the study. During the next 2 months pain disappeared in three cases and remained unchanged in five cases, only one patient had progression of pain in the treated group. Thus only 6 out of 19 evaluable patients had mainly mild pain (four grade I, one grade II and one grade III) at 1–2 months in the treated group. In contrast, in the control group aggravation of pain was remarkable in 11 of 18 evaluable patients within 2 months. Seven patients who did not have pain at the beginning complained of pain, and in four other cases pain had become worse. Thus, at 1–2 months 15 out of 18 evaluable patients in the control group displayed pain of variable intensity (five grade I, three grade II, four grade III and three grade IV). Further comparison of pain was not possible, since the majority of the patients in the control group died within 4 months.

### Time to progression and survival

In the FEMTX group, the median time to progression was 5.4 months (95% CI 3.1–11.7 months) *vs* only 1.7 months

(95% CI 1.2–2.7 months) in the control group ($P<0.0013$). This difference is illustrated in Figure 1. Similarly, a highly significant difference ($P<0.0006$) was detected in median survival between the two study groups. The FEMTX group displayed median survival of 12.3 months (95% CI 7.1–15.6 months), while the control group had a median survival of only 3.1 months (95% CI 1.6–4.6 months). This remarkable difference is shown in Figure 2. The 1 year survival rate in the treated group was 52%, and the 2 year survival rate was 24%, while 1 and 2 year survival rates were 5% in the control group.

At September 1992, 3 months after the last randomisation, five patients were alive, all of these in the FEMTX group, while all of the control patients had died. A highly significant difference ($P<0.001$, two-sided) in survivals was detected, favouring the treatment group. Owing to slow patient accrual and the conspicuous difference in patient survival, further randomisation was abandoned at this point. At the close of this analysis, May 1994, two patients in the FEMTX group were still alive, 65 + and 26 + months from the randomisation. The 65-month survivor was still in CR without any evidence of disease progression.

### *Toxicity*

Twenty patients in the FEMTX group were evaluable for toxicity. Eight patients (40%) experienced grade III/IV nausea and vomiting (Table III) which could be better ameliorated during the last 2 years of the study with new antiemetic drugs. Three patients (15%) had fairly disturbing stomatitis, and two of these and a third patient also had peeling of the skin, mainly from palmar and plantar areas. The main haematological toxicity was leucopenia: ten patients (50%) had grade III/IV leucopenia and two (10%)

**Table I** Patient characteristics

| | FEMTX (n = 21) | Control (n = 20) |
|---|---|---|
| Mean age (range) (years) | 58 (42–75) | 58 (42–71) |
| M:F sex ratio | 15:6 | 10:10 |
| Karnofsky performance status | | |
| 100 | 4 | 3 |
| 90 | 11 | 10 |
| 80 | 4 | 5 |
| 60–70 | 2 | 2 |
| Weight loss | | |
| None or <10% | 12 | 14 |
| >10% | 9 | 6 |
| Symptomatic | 14 | 13 |
| Non-symptomatic | 7 | 7 |
| Extent of disease when the diagnosis was made | | |
| Stage I–III | 7 | 5 |
| Stage IV | 14 | 15 |
| Advanced disease of primary phase/recurrent disease | 16/5 | 13/7 |
| Median diameter of largest tumour mass (range) (cm) | 5.0 (2–13) | 5.5 (2–12) |
| Locoregional/metastatic | 6/15 | 6/14 |
| Sites of metastatic disease[a] | | |
| Liver | 7 | 8 |
| Lymph nodes | 15 | 13 |
| Subcutaneous | 4 | 1 |
| Peritoneum | 4 | 2 |
| Lung | 1 | 0 |
| Other | 5 | 3 |
| Prior surgery | | |
| Biopsy only | 3 | 1 |
| Explorative laparotomy | 3 | 3 |
| Palliative | 10 | 9 |
| Curative | 5 | 7 |

[a]Many patients had more than one site of metastases.

**Figure 1** Progression-free time in the FEMTX (○) and control groups (●).

**Figure 2** Overall survival of the FEMTX (○) and control groups (●).

**Table II** Responses

| | FEMTX n (%) | Control n (%) |
|---|---|---|
| CR | 4 (19) | 0 (0) |
| PR | 2 (10) | 0 (0) |
| NC | 7 (33) | 4 (20) |
| PD | 5 (24) | 16 (80) |
| NE[a] | 3 (14) | 0 (0) |
| Total | 21 | 20 |

[a]NE, non-evaluable for response (one patient died early, evidently as a result of toxicity of the treatment; a second patient never received the study drugs owing to disturbance of renal function observed after randomisation; in the third case tumour measurements were not reliable enough to define the response).

**Table III** Side-effects[a] (WHO grade) of the FEMTX regimen

| | WHO grade 0 | 1 | 2 | 3 | 4 |
|---|---|---|---|---|---|
| Clinical[b] | | | | | |
| Diarrhoea | 15 | 0 | 3 | 2 | 0 |
| Nausea/vomiting | 3 | 7 | 2 | 7 | 1 |
| Stomatitis | 12 | 2 | 3 | 3 | 0 |
| Laboratory[b] | | | | | |
| Hb | 6 | 9 | 4 | 1 | 0 |
| Leucopenia | 4 | 2 | 4 | 6 | 4 |
| Thrombocytopenia | 17 | 1 | 0 | 1 | 1 |
| Renal disturbance | 19 | 1 | 0 | 0 | 0 |

[a]The worst toxicity observed over all cycles. [b]Twenty patients treated with FEMTX and evaluable for side-effects.

grade III/IV thrombocytopenia. Chemotherapy was stopped in one patient because of grade I renal toxicity. One possibly treatment-related death, due to sepsis, was registered. For comparison, eight cases of grade I anaemia evidently associated with disease and one case of grade II leucopenia were observed in the control group. No subjective side-effects or renal or hepatic toxicity were reported as a result of the vitamin treatment.

*Palliative measures*

We have carefully analysed all the palliative measures in the two study groups. FEMTX chemotherapy required hospitalisation for 2–3 days every month during the treatment period. Many of the patients had pain at first presentation. In the chemotherapy group pain was either relieved or unchanged, while the majority of the control patients experienced aggravation of pain within 2 months. Consequently, the use of analgesics by treated patients was unchanged or reduced and by control patient was increased. No significant differences in in-patient treatment or other supportive measures such as nutritional support, psychological support or blood transfusions could be demonstrated between the two arms. Two patients (one from each study group) underwent palliative surgery within 12 months of randomisation, one because of intestinal obstruction (control patient) and the other because of rupture of the gastric wall (FEMTX patient). Between 12 and 24 months another two surviving patients in the FEMTX group underwent palliative surgery for gastrointestinal obstruction.

### Discussion

During recent years some successes have been achieved with combination chemotherapy regimens such as FAMTX, EAP (etoposide, doxorubicin, cisplatin), ELF (etoposide, leucovorin, 5-FU) and ECF (epirubicin, cisplatin, 5-FU) in the treatment of advanced gastric cancer. With these regimens objective remission rates of more than 50% have been reported, including approximately 10% complete remissions (Wilke *et al.*, 1990; Findlay and Cunningham, 1993; Cocconi, 1994; Ellis and Cunningham, 1994). Some of these CRs have even been confirmed histopathologically.

In spite of promising high response rates achieved with these drug combinations, it has been unclear until now whether these favourable responses can be translated into prolonged survival. This study was initiated because all previous studies on gastric cancer involving a new drug or drug combination have compared results with results in patients treated with some other drug(s) or drug combination(s). In most of the previous studies the comparison group has comprised patients treated with 5-FU alone. However, there have been no randomised comparative studies on 5-FU relating 'natural' outcome of the disease to that of patients treated with the best supportive care only, without any chemotherapy.

As a treatment schedule, we selected a slightly modified course of FAMTX, a regimen reported to yield one of the highest response rates. The reason for substituting 60 mg m$^{-2}$ epirubicin for 30 mg m$^{-2}$ doxorubicin as the anthracyclin component of the scheme derived, firstly, from the knowledge that epirubicin is a less toxic drug with the same efficacy as doxorubicin. Secondly, we received information that the EORTC Gastrointestinal Tract Cooperative Group was planning a large comparative study of FEMTX and FAM regimens. We therefore decided to perform our comparative study using the same regimen. Later, however, it emerged that the EORTC study group had substituted the original FAMTX regimen introduced by Klein (Wils *et al.*, 1991). In fact, the International Cooperative Cancer Group (ICCG) is currently using FEMTX as a reference treatment in comparison with FEMTX-P, with the addition of cisplatin (P) (Wils, 1992). The aim of this study is to evaluate more precisely the role of cisplatin added to this high-dose MTX-based regimen.

Previous experiences with FEMTX in gastric cancer are limited to one phase II study with a response rate of 37% (Wils *et al.*, 1990). An *in vitro* study even suggests that in similar concentrations gastric cancer cells may be more sensitive to epirubicin than to adriamycin (Kohnoe *et al.*, 1992). If this observation could be transferred to a clinical situation, our selection of epirubicin, with a doubled dose of adriamycin compared with the original FAMTX regimen, might offer patients some advantage. Furthermore, using the same drugs with different scheduling also seems to be effective in patients with advanced colorectal cancer (Pyrhönen and Kouri, 1992).

Although the number of patients in this study is limited, our observations do confirm the fact that sequentially administered high-dose MTX and 5-FU, combined with the anthracyclin epidoxorubicin, is a highly active drug combination in advanced gastric cancer, not only in terms of objective responses but also in retarding disease and pain progression and prolonging patients' survival. Unfortunately, this study had to be stopped even before the targeted number (60) of patients was achieved, mainly because of slow patient accrual and the withdrawal of the second original research unit from the study. The analysis, however, made more than 6 years after initiating the study, demonstrated a conspicuous difference in overall survival between the chemotherapy-treated group and the control group. It was thus considered unethical to continue this study. This decision was further supported by the fact that all five patients who were alive at that time were in the chemotherapy-treated group, four of these having achieved a complete response as a result of the treatment.

In comparing the response rate of our patients treated with the FEMTX regimen, the results are similar to the combined data on FAMTX. The response rate calculated for all the randomised patients (including one patient who never received the drugs) in the present study was 29%, while the cumulative response rate for FAMTX involving 364 treated patients has been reported to be 41% (Kelsen *et al.*, 1992). The median overall survival of the present study, 12.3 months (95% CI 7.1–15.6 months), although slightly longer than in most of the FAMTX studies, was in the same range as in those studies. Nor did the median overall survival of 3.1 months (95% CI 1.6–4.6 months) for the control group differ remarkably from expectations of the natural course of advanced gastric cancer without chemotherapy. Historically, in some reports median survival of patients without chemotherapy has been reported to range from 3 to 4 months (Moertel, 1968). After a detailed comparison, the conclusion is that no difference in patient characteristics or any supportive measure except chemotherapy can explain the difference in survival in the two study groups.

Interestingly, a recent study by Murad *et al.* (1993) demonstrates very similar observations to our results. In that study patients were randomised to a modified version of FAMTX or best supportive care. In the middle of the study the randomisation was also interrupted because of strong evidence of benefit in the treatment arm. Further patients were accrued to the treatment arm, and by the end of the study 30 evaluable patients had received chemotherapy and ten supportive treatment only. The median overall survival time of the treated group was 10 months, and that of the control group only 3 months ($P = 0.001$).

The accumulating data now support the view that in patients with locally advanced or metastatic gastric cancer prolongation of survival can be achieved by treatment with chemotherapy. The particular combination used in this study seems to be of most interest. In a comparative trial by the EORTC study group, treatment with FAMTX resulted in significantly longer median survival than and similar toxicity as the widely used FAM regimen (Wils *et al.*, 1991). Another study compared FAMTX with a promising regimen, EAP (Kelsen *et al.*, 1992). Randomisation of this study had to be interrupted soon after 60 patients were collected, 30 in each treatment arm, since the EAP combination demonstrated significantly more severe toxicity, including four treatment-related deaths, and the response rate was slightly lower among patients receiving EAP than among those receiving FAMTX.

Although regimens such as FAMTX or FEMTX are still far from the optimum when the aim is cure, they can be used as comparative treatments in new developmental approaches. These combinations should also be further explored in new therapeutic strategies for gastric cancer such as pre- (Kelsen *et al.*, 1994) or perioperative chemotherapy.

**Acknowledgements**
The skilful secretarial assistance of Ms Raija Vassinen is gratefully acknowledged. This investigation has been supported by a research grant from The Finnish Cancer Society.

**References**

COCCONI G. (1994). Chemotherapy of advanced gastric carcinoma: to be completely rewritten? *Ann. Oncol.*, **5(8)**, 8-11.

CULLINAN SA, MOERTEL CG, FLEMING TR, RUBIN JR, KROOK JE, EVERSON LK, WINDSCHITL HE, TWITO DI, MARSCHKE RF, FOLEY JF, PFEIFLE DM AND BARLOW JF. (1985). A comparison of three chemotherapeutic regimens in the treatment of advanced pancreatic and gastric carcinoma: fluorouracil versus fluorouracil and doxorubicin versus fluorouracil, doxorubicin and mitomycin. *JAMA*, **253**, 2061-2067.

DUPONT JB, LEE JR, BURTON GR AND COHN I. (1978). Adenocarcinoma of the stomach: review of 1497 cases. *Cancer*, **41**, 941-953.

ELLIS P AND CUNNINGHAM D. (1994). Management of carcinomas of the upper gastrointestinal tract. *Br. Med. J.*, **308**, 834-838.

FINDLAY M AND CUNNINGHAM D. (1993). Chemotherapy of carcinoma of the stomach. *Cancer Treat. Rev.*, **19**, 29-44.

HAYWARD JL AND RUBENS RD. (1977). Assessment of response to therapy in advanced breast cancer. *Br. J. Cancer*, **35**, 292-298.

KELSEN D, ATIQ OT, SALTZ L, NIEDZWIECKI D, GINN D, CHAPMAN D, HEELAN R, LIGHTDALE C, VINCIGUERRA V AND BRENNAN M. (1992). FAMTX versus etoposide, doxorubicin and cisplatin: a random assignment trial in gastric cancer. *J. Clin Oncol.*, **10**, 541-548.

KELSEN D, KARPEH M, SCHWARTZ G, SALTTZ L, ILSON D, HUANG Y AND BRENNAN M. (1994). Neoadjuvant and postoperative chemotherapy for high-risk gastric cancer. *Proc. Am. Soc. Clin. Oncol.*, **13**, 195.

KLEIN HO, WICKRAMANAYAKE PD, DIETERLE F, MOHR R, OERKERMANN H AND GROSS R. (1983). High-dose MTX/5-FU and adriamycin for gastric cancer. *Semin. Oncol.*, **2** (Suppl. 2), 29-31.

KOHNOE S, YOSHIDA M, TAKAHASHI I, EMI Y, MAEHARA Y AND SUGIMACHI K. (1992). Epirubicin is equivalent to adriamycin in vitro against many cancer cells but more effective against gastric cancer cells. *Anticancer Res.*, **12**, 389-392.

MILLER AB, HOOGSTRATEN B, STAQUET M AND WINKLER A. (1981). Reporting results of cancer treatment. *Cancer*, **47**, 207-214.

MOERTEL CG. (1968). The natural history of advanced gastric cancer. *Surg. Gynecol. Obstet.*, **126**, 1071-1074.

MURAD AM, SANTIAGO FF, PETROIANU A, ROCHA PRS, RODRIGUES MAG AND RAUSCH M. (1993). Modified therapy with 5-fluorouracil, doxorubicin, and methotrexate in advanced gastric cancer. *Cancer*, **72**, 37-41.

PETO R, PIKE MC AND ARMITAGE P. (1977). Design and analysis of randomised clinical trial requiring prolonged observation of each patient. *Br. J. Cancer*, **35**, 1-39.

PYRHÖNEN SO AND KOURI MO. (1992). Phase II study of epirubicin sequential methotrexate and 5-fluorouracil for advanced colorectal cancer. *Eur. J. Cancer*, **28A**, 1828-1832.

PYRHÖNEN S AND VALTONEN M. (1990). Response to doxorubicin/methotrexate/fluorouracil in advanced adenocarcinoma of pancreas or biliary tract. *Lancet*, **336**, 127.

WILKE H, PREUSSER P, FINK U, ACTERRATH W, MEYER H-J, STAHL M, LENAZ L, MEYER J, SIEWERT JR, GEERLINGS H, KÖHNE-WÖMPNER CH, HARSTRIC A AND SCHMOLL H-J. (1990). New developments in the treatment of gastric cancer. *Semin. Oncol.*, **17**, 61-70.

WILS JA. (1992). Chemotherapy for gastrointestinal cancer: new hopes or new disappointments? *Scand. J. Gastroenterol.*, **27** (Suppl. 194), 87-94.

WILS J, BLISS JM, COOMBES R, AMADORI D, FOUNTZILAS G, KLEIN HO, PINTO-FERREIRA E, REIS H, VASSILOPOULOS P AND WOODS E. (1990). Phase I-II study of sequential high-dose methotrexate (MTX) and 5-fluorouracil (F) combined with epirubicin (E) [FEMTX] in advanced gastric cancer (meeting abstract). *Ann. Oncol.*, **1** (Suppl.), 43.

WILS JA, KLEIN HO, WAGENER DJTH, BLEIBERG H, REIS H, KORSTEN F, CONROY TH, FICKERS M, LEYVRAZ S, BUYSE M AND DUEZ N. (1991). Sequential high-dose methotrexate and fluorouracil combined with doxorubicin - a step ahead in the treatment of advanced gastric cancer: a trial of the European Organisation for Research and Treatment of Cancer Gastrointestinal Tract Cooperative Group. *J. Clin. Oncol.*, **9**, 827-831.